# Supplementary material for: Content-rich biological network constructed by mining PubMed abstracts
Source: BMC Bioinformatics. 2004 Oct 8;5:147. doi: 10.1186/1471-2105-5-147 (PMC528731; doi:10.1186/1471-2105-5-147)
Supplement: Additional File 2 — The original results of the above study (non-essential files are deleted to keep the file size under the limit set by BMC bioinformatics). [file 1471-2105-5-147-S2.bz2 › chilibotAdditionalFile2/dip40/513ID10344732E10815/html/CCND1_AR.html]

 


 **CCND1** and **AR** 
  
Found 122 abstracts in PubMed, retrieved 40.  
 

 What does Google say? 
 PDF only 
| .edu only 

---

**Interactive relationship** (e.g. stimulation, inhibition, etc)

**Stimulatory relationship**- **AR**  transgenic mice display increased Ras, Erk1 2, cyclin D  [ **CCND1** ]  CDK4, and cyclin E CDK2 activity and G1 S progression in pancreatic duct cells.  Ref: 12055597 Gastroenterology, 2002
- Finally, microarray analyses were performed that showed increased expression of Bcl  [ **CCND1** ]  3, an NFkappaB IkappaB factor, was correlated with AIS  [ **AR** ] .  Ref: 12538159 Ann N Y Acad Sci, 2002
- In contrast,  **AR** , a specific ErbB1 ligand, induced sustained ERK1 2 and Elk1 phosphorylation, increased cyclin D1  [ **CCND1** ]  mRNA and protein, and increased DNA synthesis activity.  Ref: 12869389 Circ ResCirc Res, 2002
- Down regulation of bcl  [ **CCND1** ]  2 protein and mRNA by DHT was inhibited by coincubation with the antiandrogen bicalutamide, an agent that competitively inhibits binding of DHT to androgen receptor  [ **AR** ] .  Ref: 12629413 J Urol, 2003
- Treatment of LY  **ar**  cells with the MEK inhibitors PD 98059, U0126, and PD 184352 led to a loss of phosphorylated ERK1 and ERK2, a reversal of nuclear p50 homodimer DNA binding, and a decrease in Bcl  [ **CCND1** ]  2 protein expression.  Ref: 12801933 J Biol Chem< MedlineTA>J Biol Chem, 2003
**Neutral relationship**- ... a sustained ERK Elk1 activation could be the major signaling cascade for  **AR**  induced upregulation of cyclin D1  [ **CCND1** ]  protein in HTASMCs.  Ref: 12869389 Circ ResCirc Res, 2003
- In addition to the effects on cell proliferation, FGF2 maintains the survival of  **AR**  PC3 clones through a positive modulation of the Bcl  [ **CCND1** ]  2 protein and down modulates  **AR**  protein expression, allowing the escape of selected clones from androgen regulation.  Ref: 12430142 Prostate, 2002
- CONCLUSION In the presence of an active  **AR** , the combined production of FGF2 and FGF BP may play an important role in the progression of prostate cancer through the selection of  **AR**  clones expressing high levels of Bcl  [ **CCND1** ]  2.  Ref: 12430142 Prostate, 2002
- Pretreatment with PD98059 suppressed  **AR**  induced cyclin D1  [ **CCND1** ]  mRNA and protein.  Ref: 12869389 Circ ResCirc Res, 2002

**Non-interactive relationship** (e.g. studied together, co-existance, homology, etc.)

- After the onset of progesterone production, Bcl  [ **CCND1** ]  2 levels decrease and Ki67 levels and androgen receptor  [ **AR** ]  expression in stromal cells disappear resulting in cell disintegration and menstruation.  Ref: 12186988 Gynecol Obstet Invest, 2002
- In contrast, expression of differentiation specific proteins prostate specific antigen and androgen receptor  [ **AR** ]  or proliferative regulatory proteins c myc, cyclin D1  [ **CCND1** ] , p27 were down regulated by hypoxia.  Ref: 12481256 Prostate, 2003
- Flow cytometric stains for antiapoptotic Bcl  [ **CCND1** ]  2 and Bcl  [ **CCND1** ]  xL showed that neither factor was well correlated with AIS  [ **AR** ] , although both were increased transiently upon T cell activation.  Ref: 12538159 Ann N Y Acad Sci, 2002
- To investigate the mechanism of the AI growth displayed by the GOF p53 mutants, Western blotting or ELISA were used to examine the expression of the androgen receptor  [ **AR** ]   **AR** , the  **AR**  regulated prostate specific antigen PSA, as well as Akt and Bcl  [ **CCND1** ]  2 under androgen depleted conditions.  Ref: 12727844 Cancer Res, 2003
- In a previously published report, we described the NF kappaB status for two murine B cell lymphoma cell lines, LY as apoptosis sensitive and LY  **ar**  apoptosis refractory, and provided evidence that NF kappaB1 p50 homodimers contribute to the expression of Bcl  [ **CCND1** ]  2 in the LY  **ar**  line.  Ref: 12801933 J Biol Chem< MedlineTA>J Biol Chem, 2003
- We found that the allele frequencies of both polymorphisms did not differ between the SID and control groups 0.65 0.35 versus 0.72 0.28 for UCP 1 Bcl  [ **CCND1** ]  I, and 0.89 0.11 versus 0.93 0.07 for beta3  **AR**  Trp64Arg in SID victims versus controls, respectively.  Ref: 12029453 Eur J Pediatr, 2002
- The expression of prostate marker genes, including 5alpha reductase, prostate specific antigen, prostatic acid phosphatase and androgen receptor  [ **AR** ] , and the anti apoptotic bcl  [ **CCND1** ]  2 gene were markedly reduced, but the transcription of apoptosis related caspase 3 gene showed a dose dependent increase in lemnabourside treated LNCaP cells.  Ref: 11837251 Life Sci, 2002
- To determine whether the UCP 1 Bcl  [ **CCND1** ]  I polymorphism and or the Trp64Arg variant of beta3  **AR**  are associated with the occurrence of SID, we determined the allele frequencies of these polymorphisms in 53 Austrian SID victims and 54 controls by nested PCR and restriction digestion using DNA extracted from Guthrie cards.  Ref: 12029453 Eur J Pediatr, 2002
- and 3 the expression of p53, pRB, Bcl  [ **CCND1** ]  2, Bax, p16, p21, p27, cyclins A, B, D1, E, p34 cdc2, and the androgen receptor  [ **AR** ]   **AR** , by western blot analysis.  Ref: 12147712 Mol Pathol, 2002
- Bcl  [ **CCND1** ]  2 and androgen receptor  [ **AR** ]  gene expression in androgen independent subclone derived from mouse androgen dependent cells.  Ref: 12197229 Cancer Invest, 2002
- We examined the impact of beta  **AR**  blockade with metoprolol on myocardial remodeling, apoptosis, pro apoptotic Fas, Fas ligand, Bax, and Bcl  [ **CCND1** ]  X S and anti apoptotic Bcl  [ **CCND1** ]  X L and Bcl  [ **CCND1** ]  2 gene expression, and Bcl  [ **CCND1** ]  X L and Bcl  [ **CCND1** ]  X S protein in post infarction HF in rats.  Ref: 12738230 J Mol Cell Cardiol, 2003
- Analysis of protein expression revealed only a weak association between Bcl  [ **CCND1** ]  2 and  **AR** .  Ref: 12098003 Neoplasma, 2002
- We conclude that adrenergic activation, such as occurs in HF, increases pro apoptotic Bcl  [ **CCND1** ]  X S expression via the beta 1  **AR** . beta  **AR**  blockade in HF reduces myocardial apoptosis.  Ref: 12738230 J Mol Cell Cardiol, 2003
- The status of antisense targeting of genes relevant to prostate cancer, including bcl  [ **CCND1** ]  2, bcl  [ **CCND1** ]  xL, clusterin, androgen receptor  [ **AR** ]   **AR**  and IGFBPs, are reviewed.  Ref: 12400997 Cancer Metastasis Rev, 2002
- The mechanisms of action may involve downregulation of the androgen receptor  [ **AR** ] , induction of apoptosis by way of inhibition of the bcl  [ **CCND1** ]  2 gene, and increased expression of p53.  Ref: 12350462 Urology, 2002
- No association was found between p21 expression and p53, bcl  [ **CCND1** ]  2, and the androgen receptor  [ **AR** ]  protein expression in bone metastases of patients with AIPC.  Ref: 11895908 Clin Cancer Res, 2002
- The status of antisense targeting of several genes, including bcl  [ **CCND1** ]  2, bcl  [ **CCND1** ]  xL, clusterin, androgen receptor  [ **AR** ]  and IGFBPs, relevant to prostate and other cancers, are reviewed.  Ref: 12643471 Curr Drug Targets, 2003
- We observed increased expressions of genes including NADH dehydrogenase, the GLUT4 glucose transporter, Na K ATPase, sarcolemmal K ATP channels, and Bcl  [ **CCND1** ]  xl in A 1  **AR**  overexpressing hearts.  Ref: 12388787 Physiol Genomics, 2002
- We investigated bcl  [ **CCND1** ]  2 and androgen receptor  [ **AR** ]   **AR**  expression in an androgen independent subline derived from mouse androgen dependent mammary carcinoma cells.  Ref: 12197229 Cancer Invest, 2002
- These data indicate that the increased expression of Bcl  [ **CCND1** ]  2 protein plays an important role in acquiring tolerance for the testosterone ablation, although it is not related to  **AR**  gene alteration.  Ref: 12197229 Cancer Invest, 2002
